# Hip-hop, identity, and conflict: Practices and transformations of a metropolitan culture

**DOI:** 10.3389/fsoc.2022.993574

**Published:** 2022-09-27

**Authors:** Luca Benvenga

**Affiliations:** University of Salento, Lecce, Italy

**Keywords:** conflict, hip-hop, metropolis, subcultures, cultural studies

## Abstract

In this paper, I expose how it is possible to investigate the hip-hop culture on three levels of analysis—historical, semiotic, and phenomenological—precisely as theorized by Cohen (1997) for his study on modern subcultures. The analysis will focus on the hip-hop of the beginning, since the middle of the Seventies and Eighties in the United States, but then it will broaden to a reflection on its diffusion and re-invented, with particular reference to the interactions with the globalization and the changes occurred in contemporary metropolis. In this sense, considering hip-hop as a subculture in the following pages, I reflect (a) on the origin of rap music, its interrelation with the Afro-American culture and the concept of blackness which it conveys. Subsequently, I clarify (b) that the art of writing, in the form of Tag, and break dance have certificated the presence of young people from the United States outskirts, and then the passage to a precise historical moment. Finally, I try to demonstrate (c) that today, a different dimension of hip-hop is implemented, whose features have changed in parallel with the transformations that have affected the metropolis and the youth cultures. These last one are increasingly hybrid and involved in a recreational, market system, where the changing nature of hip-hop allows: its enormous success, with the domain of the musical market and other assimilation processes, such as clothing, which have contributed to its mutation in a global culture, some margins of autonomy.

## Introduction and methodological note

In this paper, I expose how it is possible to investigate the hip-hop culture considering three levels of analysis—historical, structural-semiotic, and phenomenological—precisely as theorized by Cohen (1997) for his study on modern subcultures. The analysis will focus on the hip-hop of the beginning, since the middle of the Seventies and Eighties in the United States, but then it will broaden to a reflection on its diffusion and re-invented, with particular reference to the interactions with the globalization and the changes occurred in contemporary metropolis.

The English sociologist illustrates at a first level of analysis, the necessity to investigate the historical and social context where a group is rooted and develops. The structural and semiotic analysis refers to the means through which a culture reveals itself. The third level consists in an analysis of the way in which the subculture is actually experienced by its builders (Cohen, 1997, p. 57–58).

This means that, from these conceptualizations, a study of hip-hop articulated in this way should necessarily include the following:

- a reflection about geographical and territorial maps which have fostered its development (social, inter-ethnic ghettos);
- the investigation of its rituals—the subject areas of rap, writing and breaking^1^;
- the analysis, as a whole, of the symbolic values and of the meaning that these articulations represent for the main subjects of this culture.

Considering hip-hop as a juvenil culture, in the following pages, I reflect on (a) the origin of rap music, its interrelation with the Afro-American culture and the concept of blackness which it conveys. Subsequently, I clarify (b) that the art of writing, in the form of Tag, and break dance have certificated the presence of young people from the U.S. outskirts and then the passage to a precise historical moment. Finally, I try to demonstrate (c) that today, a different dimension of hip-hop is implemented, whose features have changed in parallel with the transformations that have affected the metropolis and the youth cultures. These last one are increasingly hybrid and involved in a recreational market system, where the changing nature of hip-hop allows the followings:

- its enormous success, with the domain of the musical market and other assimilation processes such as clothing, which have nevertheless contributed to a mutation of hip-hop in a global culture;
- some margins of radicalism and autonomy.

The choice to propose a study of the hip-hop culture starting from British cultural studies is hereafter explained. Anglo-Saxon analysis allow us to afford the issue from a hybrid theoretical perspective, with a particular attention to the concepts of cultural resistance, symbolical conflict, identity, and territory. Moreover, the development of the aforementioned subject, declined as follows, makes it possible to intertwine, in the second part of this paper, the different levels of analysis proposed by Phil Cohen with other more contemporary theoretical approaches, related to post-cultural studies. This provides a wider interpretative dimension from an evolutionary perspective about hip-hop.

The same Cohen, referring to specific thematic elements, as reported above, in “Subcultural Conflict and Working Class Community” (1997), states that the emergence of symbolical systems (“plastic” and “infrastructural,” in other words clothing and music, jargon, and rituals) represents the distinctive character of street groups.

Through these subsystems, the subcultural groups transfer intrafamilial conflicts outside the domestic environment, in a public dimension, where it is possible to negotiate and enhance their own identity. This occurs as a consequence of the collapse of a model of integration in the slums and the underlying subcommunitarian structure which forges the collective identity.

Through the hip-hop culture, these teenagers and young adults have used new communicational codes to react against the limits imposed on their condition. The resources they possess to affirm their identity are as follows: linguistic agitation, graffiti, symbolisms, and corporeality.

The organization of these elements, writes John Clarke, together with “activities and outlooks, which produce an organized group-identity in the form and shape of a coherent and distinctive way of ‘being-in-the-world”’ (Clarke et al., 2003, p. 54) establishes what Paul Willis has defined as homology (Willis, 2003, p. 106). The “homological relationship” between the subjective experience of the group, its values, its practices, and the use of objects evokes therefore a precise lifestyle, which I shall try to analyze below.

## Rap, blackness, and the ghetto language

The hip-hop culture of the ‘70s produces a severe conflict outside the original group—preserving by this way the relationships and social life of the group from destructive shockwaves—and one internal, oedipal, based on the affirmation of a cultural difference, as was for some classical subcultures (Cohen, 1997, p. 57 sgg.). Reflecting on the outward conflict, the hip-hop^2^ has thus generated a tension starting from the politicization of slums, through rap music used as a fundamental condition to objectify its identity in the space-time dimension of contemporary societies.

George Lapassade and Philippe Rousselot in their book *Rap il furor del dire*, from the very first pages, define rap not only as a music genre, but also “inside the ghettos where it was generated, it was soon associated to a more general attitude” (Lapassade and Roussellot, 2009, p. 13). In fact, dress styles, social stances, frequentation of places revolve around rap music, “and […] also tags, graffiti covering the walls of the city and the metro cars. This is what is described as ‘hip-hop culture’. Without this culture, rap would not exist. The first holds the second one and not vice versa” (Lapassade and Roussellot, 2009, p. 13).

The two authors, going further and deeper, illustrate the etymology of the word rap, which

comes from the American *to rap*, which means to talk, to tell, to “blurt out.” Some American philologists (Chapman, 1987) indicate a more slangy origin and associate it to the plausible abbreviation of rapid, or repartee, meaning “back and forth,” “cut and thrust” (Lapassade and Roussellot, 2009, p. 13).

The North American rap is permeated by a complex system of cultural and ethnic ties. Its roots embrace the Jamaican Toastin, the Last Poets, the Soul, and Funk music (ivi, p. 14 sgg.) On the one hand, rap is generally considered as an emancipatory reaction to the anonymity assigned to Afro-American groups by the industrial modernity. On the other hand, lyrics in rap music have been the device through which the local identity has been forged and the “Jamaican popular music has represented the somehow unavoidable spark […] after the years of black activism in America” (ivi, p. 14). Dick Hebdige writes that

rap did for poor blacks in America in the 1980s what reggae had done for the “sufferers” in Jamaica a decade earlier. It got them noticed again and it helped to forge a sense of identity and pride within the local community. Like reggae, the music later found an international audience. Additionally then, the sense of identity and pride that went along with rap became available to other people who listened to the music (Hebdige, 2004, p. 223–224).

Furthermore, by pointing out the link among rap, reggae, and the common social environment which encouraged its birth and its following development, Hebdige refers:

both reggae and rap also grew out of city slum environments. Rap started in the South Bronx of New York, which had been a mainly black and Hispanic ghetto for decades. By 1930, nearly a quarter of the people who lived there were West Indian immigrants. Additionally, most of the Spanish speakers living in the Bronx nowadays either came originally from Caribbean islands such as Puerto Rico and Cuba or are the children of Caribbean immigrants. The Cubans began arriving in the Bronx in the 1930s and 1940s and the Puerto Rican community goes back even further. There are now three million Puerto Ricans living in New York—as many as live in Puerto Rico itself. The Bronx had never been prosperous. But in the 1960s, it went into a sudden decline and by the end of the decade, it had become the poorest, toughest neighborhood in the whole of New York City (ivi, p. 224).

Rap is also and mostly connected to the “black problem.” In the Seventies, “Martin Luther King's and Black Panthers' fights seemed to be bearing fruits, and promised to the black community a brighter future.” (Lapassade and Roussellot, 2009, p. 44). If “a black middle class started to join the American middle class,” aligning to the American way for life (Lapassade and Roussellot, 2009, p. 44), the slums of Bronx, Harlem, and Wax highlighted by contrast the ambiguity of the economic integration and of the Nixon and Reagan administrations' social policies. The persistent lack of assets and opportunities to access the life standards corresponding to the American way for life leads the inhabitants of the slums, young people over all, to assume a reactive identity toward society.

Rap music, as well as describing the daily life of the ghetto, discloses the need for affirmation. The rap of the beginning is not an entertainment but a protest music (Lapassade and Roussellot, 2009, p. 48 sgg.) through which an opposition and a conflict against the American society can be expressed (the lyric of GrandMaster flash, entitled *The Message* and recorded in 1982, is significant in this respect). In some way, rap “becomes the chance to take back the word for those social groups condemned to extraneity, silence and desert.” (Petrelli, 1992, p. 87). It is an instrument which enables the subject from the slums to regain the power to speak, right that media, whose message is always unidirectional, “take off from him” (Petrelli, 1992, p. 87). Here—writes Stefano Petrelli—there is a word which hides, that of media and the mainstream. Additionally, there is […] one which unveils and consequently cures. Rap, by disclosing the existence of an underground social world, “treats” the impossibility of a live, authentic word, in the metropolis of media (Petrelli, 1992, p. 87).

These further developments frame rap even more deeply in its original culture, the blackness:

this blackness is the unique and not shareable historical experience of an entire people; it is part of the black culture of America […]. Going back to the history and the American black cultures, it seems to be universally accepted that this people have shaped two different weapons to resist the oppression and the disgraces. One is the spirituality and one is the language. From the one hand, the hope and the strength; from the other hand, the verbal agitation and the code (Lapassade and Roussellot, 2009, p. 68).

The rap of the beginnings—Seventies and Eighties—is deeply rooted in a blackness based on the biblical hope and the black activism (many texts of Public Enemy, Ice-T, and other artists will describe the life of the ghetto and will resume the unfinished fight of Martin Luther King and the Black Muslims, ivi, p. 45).

In rap music, the main role is played by the language. This is not only a way to communicate and express, but represents the attempt to put a strain on the cultural hegemony of the ruling class. Antonio Gramsci used the term hegemony

to refer to the moment when a ruling class is able, not only to coerce a subordinate class to conform to its interests, but to exert a “hegemony” or “total social authority” over subordinate classes. This involves the exercise of a special kind of power—the power to frame alternatives and contain opportunities, to win and shape consent, so that the granting of legitimacy to the dominant classes appears not only “spontaneous” but natural and normal (Clarke et al., 2003, p. 38).

Compliance and social order are reproduced not only through the organization, the management of relationships, and social interactions, but also through marks. Language, as well as objects, has a specific social connotation and is charged with cultural meaning (ivi, p. 54–55).

Rap was conceived in the ghetto, for the ghetto (Lapassade and Roussellot, 2009, p. 141). To fully understand the aesthetic of rap, we should analyze

blues lyrics and the ancient tradition of *toastin'*—an aspect of the oral Afro-American tradition consisting in telling a kind of epic story from the street with rhymes and all sorts of improvisation—and of *signifyin*'—the activity of the continuous playing with semantic neighborhoods, rhymes, and alliterations, thus creating linguistic double meanings with the often unconscious aim of deconstructing the mainstream language, to impose one's own. (u.net, 2006, p. 65).

Dozens, or the “linguistic obscenity,” the “rhymed sneer,” the “high school prank” which in blocks have similarly been the core of social relations and have organized social roles within the peer group, have their own characterization. The rap vocabulary is provided by the ghetto and the hip-hop culture (Lapassade and Roussellot, 2009, p. 79). This model of communication, claimed by Rap Brown and the Black Panthers, recalls the double essence of language, that of signified and of signifier. To analyze rap music means to observe the key relations of domination and subordination “in which these configurations stand; to the processes of incorporation and resistance which define the cultural dialectic between them; and to the institutions which transmit and reproduce ‘the culture’ (i.e., the dominant culture) in its dominant or ‘hegemonic’ form” (Clarke et al., 2003, p. 13).

The linguistic transfiguration “consists in a series of practices […] through which a person in a subordinate position tries to manage or modify the existent relation of power for his or her own benefit,” with the aim of “transforming the existing order of things or defining an open space within that order” (Pitti, 2018, p. 6).

Black culture, with its blues and popular music, has always cared about oratory and rap, in summary, represents the consequence of this folk tradition (Lapassade and Roussellot, 2009, p. 73–74): “from the first rural chants, to gospel and blues, there has always been a double level of interpretation, one for white people, who were pleased to listen to the ‘bravo nigger’, and one for black people who could understand the message” (ivi, p. 76).

A political, existential message will use oratory and new aesthetic values, like writing.

## Writing and identity. From rumble to bombing

Hip-hop presents itself as a strong antagonist philosophy developed in the ghettos of U.S metropolis (Barile, 2019, p. IX). From the beginning, we have seen that the leading force of rap music represented the distinguishing mark of this culture, whose set of languages includes, besides Rap (or Mc'ing), Writing, Dj'ing (or Turntablism), and break dance. The art of writing has a significant development with the Rats in the Street to whom Malcolm X refers in some of his famous speeches pronounced between the end of the Fifties and the beginning of the Sixties (Naldi, 2020, p. 48). Taki 183, Cornbread, Julio 204, just to mention the pioneers of writing (Castleman, 1982, 2004), or better still of “lettering” to be more precise. (ivi, p. 49), are among the first Rats in the Street who choose to assume a new identity to record their passage through scratched signs on the wall (Naldi, 2020, p. 48).

These processes of individual and social identity building, referred to writing, are present in one of the first European reflection on the phenomenon proposed by Jacky Lafortune, who outlines:

le tag est une signature individuelle réalisée avec un lettrage particulier. Le 21 juillet 1971, le New York Times consacre son premier reportage au tag. Les exploits du taggeur surnommé Taki 183 y sont relatés. Comme Roger Mettalic Avau le remarque, “le New York Times en tête donne un sérieux coup de pouce [aux taggeurs] en parlant d'eux. Une nouvelle forme de graffiti était née [...]. Quoi de plus facile, la nuit tombée de se glisser dans un dépôt du subway et de reproduire son tag en grands caractères sur les rames? Des centaines de tags vont ainsi déferler [...]. On entre de plain-pied dans l'ère'du moi je', le désir de sortir de l'anonymat [...]. Ce qui différencie le tag du graffiti soixante-huitard [en Europe], c'est qu'il s'agit, non plus d'une revendication mais d'une affirmation” (Lafortune, 1993, p. 74).

Also according to Petrelli, with the Tag, the writer develops a presence on his own. In fact, this “graphic and linguistic explosion” (Naldi, 2020, p. 48), reflected on the increasing occupation of portions of urban territory—particularly in the outskirts—offers the possibility to express and perceive oneself as an active subject:

[tags can be seen] as an attempt—made by the author—to Become Visible in a metropolis whose social politics, and cultural practices, tend to make invisible even wider social groups. It is not only an economical, social, cultural marginalization: it is a *physical* one. It is physically that people in the Hispanic or Afro-American outskirts in the United States […] perceive this exclusion. They, […], feel that today, the action of mass media, which produce signs and therefore of meaning, is relevant – without foreseeing their presence. Against this kind of production, tags [impose] themselves as a signature-presence (Petrelli, 1992, p. 87).

Young people from the outskirts, excluded from the educational and work system, are in search of redemption. Thus, they “try to establish their own identity developing a culture of the negative. The positive values of mainstream culture are therefore distorted and inverted to better fit in a philosophy of dissent and protest” (Gatti, 1983, p. 136).

The search for a sense of identity is achieved in the concept of gang, which, in the Seventies in the US, was already growing to include hundreds of young members, whose main activity revolves around the *turf*, the “territory under their dominion in a constant need for defense and expansion” (Gatti, 1983, p. 135). According to Gatti

The gang is the collective reply to the misery and the troubles of the so-called “high-risk neighborhoods,” the urban areas with the highest crime rates, overpopulation, child mortality rate, and epidemics (ivi, p. 136).

The control over the territory exerted by the gang, in legislative (the law of the strongest), economic (illegal activities), and cultural terms (Afro-American, Hispanic, Italians, etc.), is implemented through the involvement in violent physical confrontations

or gang war, an activity which was even feared by the gang leaders […], the only way to gain respect for the other gangs. To increase one's individual reputation, there is the *fair one*, the loyal fight between two selected members of rival gangs [loyal because it is not possible to use weapons or any help from their fellows] (ivi, p. 136).

Nevertheless, it is with the first tags realized by a young of Greek origins, Demetrius aka Taki 183 (in 1971)—the numeric code refers to his house number—that the phenomenon of the gangs, the system of symbols and signifiers which mostly inspired the action of the Turf, assumes a new form:

in the new era opened by Taki 183, the gang war is no more called rumble, but a cross-out, an erasure war, not implying weapons but cans of spray paint. It is no longer the stronger or more popular gang to win, but the one writing more and better. The gash perceived as a war declaration consists in smearing other gangs' graffiti. The reply of the enemy triggers a conflict, which can last for weeks or entire months (ivi, p. 138).

Tags become for young writers the opportunity to be noticed, to move and escape from their turf, alone or together with the gang. “From the poorest outskirts, hordes of young people invade the city center and hit each train and stop with their spray paint, their enormous brushes and their guerrilla warfare techniques” (ivi, p. 137).

According to Castleman (1982), the elements in common between the art of writing and the phenomenon of gangs are to be searched in the practice of illegal activities, which require ability, skills, and practical knowledge; but also in the use of heteronyms to escape the repression of the police, and most of all, they share the power to affirm their identity (in the case of graffiti by infringing on the private or public property).

In a few years, thousands of young people “attracted by the power of ubiquity” (Gatti, 1983, p. 137) hit, with a bombing action, hundreds of train cars, and metro stations. As time goes by, with an extraordinary expansion, both quantitative and qualitative, the first tags become a genuine urban art, with different forms and styles:

so tags, born from acronyms and signatures, thus as a form of *writing*, become real and autonomous artistic forms, complex compositions planned and then realized (pieces). Letters dilate in space and fill in color creating large-scale images (*masterpieces*). Characters (*block letters*) swell up (*bubble style*), acquire an additional dimension (*3d style*), and finally loose their function becoming deliberately unreadable shapes (*wild style*) (Dal Lago and Giordano, 2018, p. 39).

An important individual and collective participation is balanced by a sensational reaction of displeasure on the part of the institutions, which conceive graffiti as a mere act of vandalism. The war against graffiti, inaugurated by Lindsay, the mayor of New York city, will last for decades and will cost the community several hundred million dollars (Castleman, 2004, p. 22 sgg.; Mansbach, 2013). Only in the first Seventies, the police arrested more than 1,500 young people, most of all minors from the suburbs. Anyway, the years when Taki becomes a popular hero, and thousands of his peers follow his example, are the “exciting time of the hip-hop culture,” linked to the Afro-American and Latinos' claims, “in other words to the attempts of taking the floor from the voiceless in the American society of the time” (Dal Lago and Giordano, 2018, p. 38). The war against graffiti can then be interpreted as a pretext to contain the demands of generations of excluded, articulated through rap music, writing and, as we will analyze in the next paragraph, also through dance, with break dance. About this war against the entire hip-hop culture, Mansbach remarks:

those stakes become clearer when one examines law enforcement's public profiling of graffiti writers. They were described as “black, brown, or other, in that order,” and vilified as sociopaths, drug addicts, and monsters. This was a fight over public space, and we would do well to remember that at the time the fight began, teenagers were also being arrested for break dancing in subway stations, and throwing un-permitted parties in the asphalt schoolyards of the Bronx. Taken collectively, these three activities also represent the birth of hip-hop, the single most influential subculture created in this or any country in the last half-century (Mansbach, 2013).

## Break to dance. styles, fashion, mainstream

If on the one hand, hip-hop culture “has found in urban spaces a fundamental place of production and reproduction since the beginning,” then on the other hand, some areas of the city “have been deeply influenced by hip-hop practices and its performance.” Owing to the fact that

the city is recorded in rhymes and infiltrates in *block parties*, hip-hop snakes the corners of the streets, is inscribed on the walls, and re-signifies the spaces: a biunivocal relation in which none of the two directions can be neglected (Giubilaro and Pecorelli, 2019, p. 24).

This re-signification of the urban space, about which the authors speak, is also part of the reflection of Gatti, who focuses on the places sharing break dance, writing, and rap music as ways of expression. Breaking requires a public arena, as well as “other forms of youth culture – writes Gatti. Differently from other dances, it wasn't born in night-club, but in the street, in the park and in the metro station, of course” (Gatti, 1983, p. 141).

We have abundantly explained (above) that the hip-hop culture, through its disciplines, originates from a need for subjectivation and participation. The transfer in the spare time context of those pleasures forbidden in institutional places (school and work) is a reply to a specific social and economic condition, as the consequence of a structural violence. Hip-hop is a means to bring the knowledge, or “self-awareness,” cultural independence, and autonomy to the surface. It is the search for a political collocation, aimed at weakening the prevailing power relations, together with rap, street style (graffiti art), body language (break dance) to become the mouthpiece of new individualities and a social status, otherwise denied.

Like a graffiti artist makes use of his name to build an identity, at the same way the breaker uses his body—outlines Martha Cooper. “Break dance is a genuine celebration of flexibility and sensuality of the male teenager body.” The speed of movements and the smoothness of drawings are essential both in breaking and in graffiti art. What matters is mostly the degree of difficulty. For this reason, like in graffiti and rap music, breaking is an ever-changing form of art. The purpose is, in fact, the development of new techniques and styles to overcome the others and affirm one's supremacy. What is at stake is the social position in the community, which often represents for the young inhabitant of the ghetto everything he owns. (ivi, p. 141).

If through bombing, it is possible to exorcize the rumble, break dance avoids the reference to bombing with the individual and collective involvement in creative works, instead of criminal activities (Cristante, 1983, p. 37). In fact, according to Holman (2004), “the first real breakers were the gang members of Black gangs in the Bronx in the late 60s, early 70s. These guys did a dance called the Good Foot, from James Brown's record of the same name. The Good Foot was the first freestyle dance that incorporated moves involving drops and spins and resembled the beginnings of breaking” (ivi, p. 36). So, the “acrobatic and athletic ritual embeds some elements of ballet and fight, becoming an artistic substitute of a physical confrontation” (Gatti, 1983, p. 141).

With the break dancing phenomenon, exploded in the Eighties and Nineties, the B-boys (or Fly-Girl) acquire a visibility (also in the media), mostly identifying in a process of stylistic creation, or in the rearrangement and re-contextualization of objects and communicating “new meanings, inside a system of values which already includes connotations sedimented from the origin and connected to those objects” (Clarke, 2003, p. 205). By expropriating and re-appropriating in this way cultural meanings (Clarke et al., 2003, p. 76), the B-boys were used to wear “suits, sport shoes, caps, chains, and ornaments [like rappers]: a clear sign of fetishist re-appropriation of an item which used to be read as an expression of the cultural subordination of the ‘black nation’ since colonialism” (Barile, 2019, p. IX).

Style objectifies the image that the group has of itself. It is clear therefore why the subcultural group is interested in a certain kind of objects and not in others.

In this respect, Clarke writes:

The important point here is that the group must be able to recognize itself in the more or less repressed potential meanings of particular symbolic objects. This requires that the object in question must have the “objective possibility” of reflecting the particular values and concerns of the group in question as one among the range of potential meanings that it could hold. It also requires that the group self-consciousness is sufficiently developed for its members to be concerned to recognize themselves in the range of symbolic objects available. This developed self-consciousness both in terms of its content (their own self-image, etc.) and in terms of its orientation toward symbolic objects is the means through which the style is generated. The selection of the objects through which the style is generated is then a matter of the homologies between the group's self-consciousness and the possible meanings of the available objects. (Clarke, 2003, p. 179).

To the uptown culture, the first name given to hip-hop—being born in Uptown Manhattan—was often associated with the so-called sky fashion, the use of glasses, coats, and ski hats (u.net, 2006, p. 41). This clothing, originally very expensive but in use by under-class people thanks to subjective intuitions, caught the attention of fashion magazines (such as, among others, East Village Eye).

At the same time, in the middle of the Eighties, an increasing interest from the part of mainstream media was mostly addressed to break dance dancers. Sally Banes comments:

although breaking is the newest part of hip-hop culture, it's the part that has made hip-hop, a media obsession. Then, 5 years ago, the only people who had ever heard of breaking were the kids in New York's ghettos who did it. They didn't even have a definite name for the form—they sometimes called it “breaking,” but they also referred to it as “rocking down,” “b-boy,” or just “that kind of dancing you do to rap music.” By 1980—when the form had already been around for a few years—they weren't even very interested in it anymore. This kind of dancing was a passing fad, they felt, that would soon be replaced by roller disco. But history was to prove them wrong. Not since the twist, in the early sixties, has a dance craze so captured the attention of the media (Banes, 2004, p. 13).

Although long, we report another quote from Banes, which provides a clearer idea about the impact of break dance in cultural industry and entertainment. This suggests the beginning of an influence of globalization on the hip-hop culture:

By 1984, only a hermit could *not* have known about breaking. It had arrived, not only in the United States but also in Canada, Europe, and Japan. Breaking had been featured in the 1983 Hollywood film *Flashdance*, the independent hip-hop musical film *Wild Style*, and the documentary *Style Wars* (which aired on PBS), served as the inspiration for the 1984 films *Breakin*' and *Beat Street*, and was rumored to be the subject of fifteen forthcoming Hollywood movies. Countless how to books and videos had hit the market. Breaking had been spotlighted on national news shows, talk shows, and ads for Burger King, Levi's, Pepsi-Cola, Coca-Cola, and Panasonic. In total, one hundred break dancers heated up the closing ceremonies of the 1984 summer Olympics in Los Angeles. In addition, Michael Jackson had given the form national currency. Breaking made the cover of *Newsweek* in 1984. Newspapers all over the country regularly carried stories on its latest ups and downs. The paradox emerged, as you flipped the pages of the *Washington Post* or the *Los Angeles Times*, that break dancers who would come up in the ghetto were banned from city streets and shopping malls for causing disturbances and attracting undesirable crowds, while at the same time, middle-class housewives and executives could learn to break dance in their spare time at classes proliferating throughout the suburbs. Doctors added to the form's acceptability by giving medical advice on how to survive it unbruised. In addition, the *New York Times* began using breaking as a metaphor even in articles that had nothing to do with hip-hop (Banes, 2004, p. 13).

Hip-hop goes hand-in-hand with the transformations of styles, youth culture and urban space. These changes have given new life to the content of rap songs and have encouraged the street art exposition in galleries and the creation of a Decalogue of acrobatic steps to be hanged in dance schools. This has partially emptied hip-hop of its social context and has consequently driven it away from its significance. Nevertheless, with several disciplines—and rap in particular, as we will analyze afterward—young people of the end and the beginning of the century still explore creativity and identity through a new relationship with the territory and the cultural industry.

## The transformation of youth cultures and of street style. The two souls of hip-hop

It has been frequently outlined through the study of its origins, of the three main areas of hip-hop culture and of the link with the ethnic group and the reference territory, that the place of expression for the young generations has always been the metropolis. Articulated in micro-forms and relegated to the borders of the urban space, these expressions of identity concern social and territorial groups often represented by the mainstream media as problem generators. It is a conflict which refers to forms of typical antagonism (cultural endurance) characterized by new elements and that, in a short time, has acquired transnational contours through the media.

Furthermore, this internationalization of the conflict will be fostered by the rapid development of the Net and the deterritorialization of cultures, factors which will influence the socio-economic structure beyond the lifestyle of the street groups of the Nineties.

With the development of sophisticated systems of information, in Western countries at least, the factory and the productive process—as a scene of struggle and social aggregator—have given way to communication and new lifestyle. Forms of traditional living have melt, impoverishing stable relationships, district economies, and implicit mechanisms of social safety nets. The impact of these transformations on specific social groups (privatization of needs, functionalization of some urban areas to the reproduction of low-paid workers, gentrification of districts, such as Gracia district in Barcelona or the ghettos of Los Angeles) compels us to focus on the restrictions imposed on street-based entertainment activities. This has encouraged the emergence of a new institutional framework, which has represented the end of Foucault's disciplinary society (with the comeback of a pre-bourgeois system of prevention, with still clearer physical boundaries), determining the advent of forms of social control not aimed at the creation of bodies functional to the productive cycle, but to the constrictive limitation of the surplus of human groups (De Giorgi, 2002), part of a power dynamic which does not guarantee anymore a social citizenship (De Giorgi, 2002).

Thinking the coexistence of safety policies in metropolis, clearly visible in the technical rationalization of the physical space, with socialization processes explicated by consumption, is considered a precondition to the production of a feasible plan of analysis for the study of youth cultures. It is starting from the acceptance of this new definition of relations and interactions within the social system, that we want to stress the individualistic impulse of new generations, and the commercial co-optation of street style, as a consequence of the “increasing collusion with the brand system” (Barile, 2019, p. IX). “Both of all are dynamics which cross the rap arena (and mostly trap) characterizing representations, lyrics, and professional choices of its main actors in a more or less explicit way” (Cuzzocrea and Benasso, 2020, p. 343):

the aesthetic intentions and the demands created in the contexts of contemporary youth arenas are definitely more focused on politics of existence than of resistance, thus aiming at the social recognition of a self-sufficient personal identity, which defines itself as singular, authentic, creative, and free (Ferreira, 2016, p. 68, cited by Cuzzocrea and Benasso, 2020, p. 243).

Nevertheless nowadays, analyzing collective behaviors from a cognitive perspective, discussing class and territorial affiliations (the street, the pub, the square, and the corner shop), could result in a timelessness in the time of *sharing*, of *fake*, of the absolutization of cyberspace and of the performative subject (from work to consumption).

In the last decades, a set of completely new living conditions have firmly established their position. They have benefited a process of osmosis among “image-consuming” cultures, no more identifiable in a changeless collective subject, but inspired to a hybrid collectivity (Pohlemus, 1994) based on an aesthetic peculiarity. At present, the countless articulations of youth cultures do not allow the understanding of what is meant by radicalization of indicators in performative subcultures, in proletarian suburbs, in the critical and reflective elaboration of aestheticization, analyzed as means of conflict together with a class and generational factor.

In this context, it is possible to observe an objective condition of accelerated cut-up on the part of the new generations, inscribed in a contingency where social and political borders are no more valid. The dividing line among opposed cultural genres begins to crumble with gothic cultures, where the antithetical visual dualism between working class subculture—referring to Skinhead, Mod, and Ted styles—and middle-class counter culture (Hippies, Provos, etc.) splinters and all those forms of re-enchantment based on new forms of religiosity, often new age, dematerialize.

From the Eighties and Nineties, the classic concepts of subculture and street style enter into crisis because of “two simultaneous processes: on the one hand, the increasing value of communication, which rapidly circulates the signs of various ‘stylistic isles’, fostering hybridization and crossover phenomena in two different ways of style surfing and of *sampling'n'mixing*” (Barile, 2019, p. IX); on the other hand, the commercialization of a young lifestyle is always more sensationalized by marketing requirements. All these aspects have in some way also influenced hip-hop.

From the block of flats in Sedwick avenue—in the Bronx of 1973—, during an event organized to encourage socialization among teenagers living in the neighborhood and collect money to buy school uniforms (Nexus, 2020, p. 20 sgg.), a popular history started and still today, after more than 40 years, holds together in a global but binary dimension, thousands of territorial realities. In fact, whereas at the beginning of the Seventies, the militant soul of hip-hop was prevailing and then, during the Eighties, a certain balance between the strong political connotation and the playful soul was reached, and finally, during the Nineties,^3^ the commercial aspect became the predominant one (Barile, 2019, p. IX).

Anyway, even if the commercial soul “will become a distinctive sign of this style” (Barile, 2019, p. IX), “hip-hop, declined in rap, in some areas of the outskirts is simultaneously place of agency and personal success and a means for negotiation of spaces and collective identities” (Giubilaro and Pecorelli, 2019, p. 24), and also daily practice of activism. In this respect, Giubilaro and Pecorelli write that

The diffusion of rap and its commercialization have made the genre so articulated and heterogeneous to make it difficult to state anything except for analysis based on the singularity of its practices and manifestations […]. Furthermore, as well as any other cultural expression, rap is the product of complex hybridizations among hegemonic and counter hegemonic practices, which sometimes can coexist inside a same performance, lyric, or rhyme (Giubilaro and Pecorelli, 2019, p. 25).

If on the one hand, the commercialization of rap music “has permitted the diffusion of the hip-hop culture at an international level” (Neal interviewed by u.net, 2006, p. 37), on the other hand, this cooptation from the cultural industry has transformed it in an accessible product. In this way, it occurs what Clarke has defined as the “defusion” of a life style, concept which can be understood within the usual opposition between reappropriation from the bottom or from the top (thus from the mass media) of a culture:

“defusion” we mean that a particular style is dislocated from the context and group which generated it and taken up with a stress on those elements which make it “a commercial proposition,” especially their novelty. From the standpoint of the subculture which generated it, the style exists as a total lifestyle; *via* the commercial nexus, it is transformed into a novel consumption style. Typically, the more “acceptable” elements are stressed, and others de-stressed. (Clarke, 2003, p. 188).

Nevertheless, the hip-hop of the new millennium has also succeeded in both taking the transformations and saving spaces for authenticity (Pedretti and Vivan, 2009, p. 168). From Europe to Americas, from the French Casey, Kaaris, NTM, who participated and “commented” the riots in Parisian banlieue of Clichy Sous Bois, to the Chilean Ana Tijoux and the Doblecero, hip-hop presents a “transnational” (Meghelli, 2012; Gardner, 2014) version, with the purpose of reinforcing social nets among people living at the edges of the great productions, or precariously. Hip-hop resumes, one more time, the concept of territory (metropolitan quarters), of ethnicity, of youth, and of the self who realizes himself in a collective subject assuming the will of a critical and self-reflecting position about his own condition.

From these examples, it can be noticed that the existence of spaces for autonomy—understandable within what we can define the proliferation of styles and distinctive signs, typical of contemporary youth cultures (Pohlemus, 1994; Muggleton, 2000)—still gives the possibility to the hip-hop culture to speak about social injustices, poor districts, and power more than a mere *bling bling*. This happens inside the entertainment industry, exploring those channels (especially digital) whose aim is to promote an artificial image of the hip-hop movement.

The hip-hop “‘beats his time’—writes Riccardo Pedretti—proposing a critical analysis of contemporary society which follows a harsh path and full of pitfalls using its same means in a subversive manner” (Pedretti and Vivan, 2009, p. 169).

## Conclusion

The socio-historical contexts, and the rituals of the hip-hop culture which we have discussed in these pages, have permitted a comparison among the socio-anthropological complexity of young hip-hoppers' cultural productions, referring to Cohen's three levels of analysis. Showing a continuity with the concepts of the English sociologist, the key points of the analysis have been represented by a generational culture, born and grew up in ethnic ghettos (corresponding to the historical level), by the different areas of hip-hop (the semiotic level) and the lifestyles (sense of territory, affiliation, solidarity, creativity, etc.), referring to the phenomenological level. In this conceptual frame, a reflection has been developed about subjective and collective affirmation processes, created since the Seventies and the following decades, starting from the outskirts of U.S. metropolis. Subsequently, these aspects have enabled us to reason about the means through which we can understand the transformations of street style, after fundamental changes in the metropolis and the youth culture.

Starting from a territorial perspective, it has been possible to understand an action collocated in a wider space and provided by communicational codes which, crossing contemporary world, have overwhelmed several youth groupings. Their reaction to the continuous socio-economical conflicts in the metropolis has been lived in other places than those imposed by the dominant codes (school and work), and in more symbolic subsystems, “infrastructural” and “plastic” (objects, clothes, music, and places attended).

These processes of affirmation, which have often had the less social protection among their protagonists—at the beginning of the hip-hop culture over all—have revealed themselves in spatial practices of re-appropriation of the neighborhoods in a “street grammar” as arrogant as diversified.

With reference to the U.S. context, the young subject has started to impose himself since the de-fragmentation of the (social and cultural) self-perception, in those areas overwhelmed by a runaway urban renewal (which was also in all respects an anthropological and cultural change). In the years of a large-scale gentrification since the Sixties, an irreplaceable crack began to reproduce in the secular balance of traditional economic and family structures. From their contradictions, a new youth symbolic and cultural universe took shape.

The hip-hop subculture has thus become an opportunity for redemption and for the conquest of a status, modifying the processes through which identity is shaped and changing the modes of (extra-familiar) socialization of the new generations, focused on the construction of their personal histories by subverting social routines.

In a retrospective evaluation, hip-hop has been determined by the convergence of several factors. Social contingencies, cultural trends, and endogenous variables are peculiar elements to understand the features, the complexity, and the increasing reaction to the hegemonic culture.

Finally, changes in the hip-hop culture of the last decades have been mentioned, and the unbalance toward the commercial soul more than toward the political one has been observed, even if it has not consisted in a loss of its original force. This is given to the fact that “the underground version continues to carry ideas, besides it enables a social protest and represents an educational instrument […], so it can significantly enter the opinion-making processes, particularly among young people […]” (Privitera, 2016, p. 72).

In addition, these changes have to be contextualized inside a larger break, which affects youth culture and the generations born and grew up in the society of consumerism and globalization. In this case, street style cannot be related to a relationship of power, to social classes, and it is not the epiphenomenon of economic structures which forge experiences of marginality and realize some reactive choices (Corchia, 2017, p. 308). In fact, as Luca Corchia illustrates in his analysis of youth contemporary identities, “cultural affiliations [are] multiform and changing, some partially or totally opposed to the *mainstream*, others, partially or totally adherents to the hegemonic culture”. Paraphrasing Bennett, Corchia continues commenting on “relational nets characterized by fluid temporal borders and ‘floating’ forms of belongings”, in which “the existence develops through a fragmented series of real and virtual spaces, in which identities and roles dependent on the interactions of here and now are explored, before collocating in new contexts and acquiring still different roles and identities” (ivi, p. 311).

## Data availability statement

The original contributions presented in the study are included in the article/supplementary material, further inquiries can be directed to the corresponding author/s.

## Author contributions

The author confirms being the sole contributor of this work and has approved it for publication.

## Conflict of interest

The author declares that the research was conducted in the absence of any commercial or financial relationships that could be construed as a potential conflict of interest. The reviewer VF declared shared research group Ais- Associazione Italiana di Sociologia- section: Sociology of Sport with the author LB to the handling editor.

## Publisher's note

All claims expressed in this article are solely those of the authors and do not necessarily represent those of their affiliated organizations, or those of the publisher, the editors and the reviewers. Any product that may be evaluated in this article, or claim that may be made by its manufacturer, is not guaranteed or endorsed by the publisher.

## Footnotes

1 The *hip-hop architecture* is also composed by other practices, such as Djing, which I shall not explore in this paper, because it is considered part of the sound system (soundtrack consisting in the plates to the disks and the microphone). In fact, the rapper must mark his elocutions (timing) in time with the dubbing line, produced by the Disc Jockey (Lapassade and Roussellot, 2009, p. 154–155).

2 Before proceeding with the analysis, it is important to clarify that the prevailing models of reference of the hip-hop culture mostly reproduce the features of hegemonic masculinity, just as theorized by Connell (1995), even if over time a new space for not hegemonic masculinities is developing (see for example Frank Ocean). Territorial and/or ethnic relations are homosocial and, as occurring in classical subcultures (Hall and Jefferson, 2003), the presence of women, at least in the hip-hop of the beginning, is marginal and subordinate. Nevertheless, female artists or women-only groups have emerged over the years, from music to break-dance. Their role has become increasingly central and they have raised questions connected to gender relations in the hip-hop culture. Anyway, given the vastness of the subject, I shall not include the analysis of gender models in this paper. Given the obvious impossibility to provide a complete bibliography, I shall therefore just mention: Morgan (1995) and Schloss (2009).

3 According to Grag Tate, the TV channel MTV plays a key role in this sense: “it has been the functional means for the diffusion of these videos at a global level. Many teenagers got to know rap through this channel. While the hard-edged and lean sound of the hip hop productions captivated increasing market shares, the capitalistic system began to take action to co-opt this culture, diluting it to make it more appealing to a white public. Productions started to be imposed from the top instead than from the bottom, by the artists' creativity […]. From an aesthetic point of view it has had an impact similar to the revolution brought by jazz and bebop, whilst from a commercial point of view it obtained the same popularity than soul and funk (Tate interviewed by u.net, 2006, p. 137).
